# Comparative Analysis of Zearalenone Effects on Thyroid Receptor Alpha (TRα) and Beta (TRβ) Expression in Rat Primary Cerebellar Cell Cultures

**DOI:** 10.3390/ijms19051440

**Published:** 2018-05-11

**Authors:** David Sandor Kiss, Eniko Ioja, Istvan Toth, Zoltan Barany, Gergely Jocsak, Tibor Bartha, Tamas L. Horvath, Attila Zsarnovszky

**Affiliations:** 1 Department of Physiology and Biochemistry, University of Veterinary Medicine, 1078 Budapest, Hungary; kiss.david@univet.hu (D.S.K.); toth.istvan@univet.hu (I.T.); barany.zoltan.balazs@univet.hu (Z.B.); jocsak.gergely@univet.hu (G.J.); bartha.tibor@univet.hu (T.B.); 2 Department of Animal Physiology and Animal Health, Faculty of Agricultural and Environmental Sciences, Szent István University, Páter Károly u. 1, H-2100 Gödöllő, Hungary; ioja.eniko@mkk.szie.hu; 3 Department of Comparative Medicine, Yale University School of Medicine, New Haven, CT 06520, USA; tamas.horvath@yale.edu

**Keywords:** zearalenone (ZEN), thyroid receptor α (TRα), thyroid receptor β (TRβ), primary cerebellar neurons

## Abstract

Thyroid receptors play an important role in postnatal brain development. Zearalenone (ZEN), a major mycotoxin of Fusarium fungi, is well known to cause serious health problems in animals and humans through various mechanisms, including the physiological pathways of thyroid hormone (TH). In the present study, we aimed to investigate the expression of thyroid receptors α (TRα) and β (TRβ) in primary cerebellar neurons in the presence or absence of glia and following ZEN treatment, using quantitative reverse transcription-polymerase chain reaction (qRT-PCR) and Western blot. Primary cerebellar granule cells were treated with low doses of ZEN (0.1 nM) in combination with physiologically relevant concentrations of l-thyroxine (T4), 3,3′,5-triiodo-l-thyronine (T3) and 17β-estradiol (E2). Expression levels of TRα and TRβ at mRNA and protein levels were slightly modified by ZEN administered alone; however, along with thyroid and steroid hormones, modelling the physiological conditions, expression levels of TRs varied highly depending on the given treatment. Gene expression levels were also highly modulated by the presence or absence of glial cells, with mostly contrasting effects. Our results demonstrate divergent transcriptional and translational mechanisms involved in the expression of TRs implied by ZEN and hormonal milieu, as well as culturing conditions.

## 1. Introduction

Zearalenone (ZEN, also known as F-2 toxin), a potent, non-steroid estrogenic mycotoxin is produced by a variety of Fusarium fungi found worldwide. As a regular food pollutant, it occurs along with other mycotoxins in products such as nuts, corn, rice, and several other cereals contaminated with these toxins during harvest or storage [1]. Zearalenone is rapidly absorbed after ingestion and is extensively metabolized by intestinal cells [2]. The two main metabolites of ZEN are α-zearalenol and β-zearalenol with various chronic and acute effects in humans and animals [1,3,4]. By binding to nuclear estrogen receptors (ERs), ZEN and its two active metabolites have a strong estrogenic potency [4] and therefore are mostly implicated in disorders related to the reproductive system [5,6,7,8]. Several studies demonstrated the DNA-damaging effect of ZEN via DNA adducts, fragmentation, and chromosome aberrations in different cell-lines [9,10]. ZEN involvement in the disruption of endoplasmic reticulum homeostasis and the activation of mitochondrial apoptotic processes was also demonstrated [11,12]. Very few studies investigated the effects of ZEN on the central nervous system and/or on neurotoxicity [13,14]. In human neuroblastoma cell lines, a marked suppressive effect on neuronal gene expression and ZEN-induced reactive oxygen species (ROS) formation was observed [15]. Studied in different cell lines, ZEN exhibited competitive inhibitor properties on aromatase or CIP19, inhibiting the biosynthesis of estrogen [16]. On the other hand, other studies reported the positive effects of ZEN on hippocampal neurogenesis and cognitive improvement in ovariectomized mice and rats, similar to those caused by 17β-estradiol (E2) [17,18,19]. The neuroprotective effects of ZEN metabolites were also demonstrated in cultured primary hippocampal neurons exposed to amyloid-β peptide (25–35) [20]. 

In the present study, we sought to investigate the impact of ZEN—alone or in combination with thyroid hormones (THs) and E2—on thyroid signaling and gene expression patterns in rat primary cerebellar cell cultures. Primary cultures from the cerebellum are widely employed for in vitro studies since they provide an excellent model to study neuronal development and the expression patterns of several receptors, thus, applied for molecular and cell biological studies [21]. Moreover, cerebellar granule cells are also generated postnatally and can be kept in culture as a homogenous, mature, neuronal population [22]. The effects of THs alone or in combination with E2 on TR and ER mRNA and protein expression in primary cerebellar cultures were previously investigated in-depth by our research group [23]. By controlling various biological processes (metabolism, bone growth, etc.), THs play an important role in brain development, especially during perinatal periods [24]. THs exist in two major forms, the main active 3,3′,5-triiodo-l-thyronine (T3) and its precursor, thyroxin (T4) converted by 5′-deiodinase within target tissues [25]. In the central nervous system THs have a differentiated transport favoring T4 whereas conversion of T4 is carried out in glial cells especially in astrocytes (and tanycytes), by type-2 deiodinase (D2) already present in glial cells at fetal stage [26,27]. This differentiated transport and conversion of T4 in glial cells proves to be a major hotspot for the causes of neurodevelopmental disorders, demonstrated in different animal models [28,29]. Moreover, THs have a more complex non-genomic effect on astrocytes and extracellular matrix, interacting with different signaling pathways, thus impacting neuron-astrocyte interactions and affecting brain development and function [30,31,32]. THs modulate the proliferation and differentiation of glial cells [33,34,35], migration of granular precursor cells, development of dendritic arborization of Purkinje neurons, as well as maturation of interneurons [36]. A differential expression pattern of TRs and ERs in neuronal cells and a potential modulatory role of glia in THs and E2 signaling was also proposed and shown by our group [23]. The impact of various endocrine disruptors alone or in combination with THs and steroid hormones were also investigated by our laboratory [37]. Other groups also showed that different chemical disruptors, present in low concentrations in food and water, exert their toxicity by interfering with T3 signaling [38]. In this study, the modulatory effects of ZEN, as a known endocrine disruptor, on the expression pattern of the two main TR receptors—TRα and TRβ—at mRNA and receptor protein levels were investigated. 

## 2. Results

### 2.1. Expression of Thyroid Hormone Receptor Alpha (TRα)

#### 2.1.1. TRα mRNA Expression

**Figure 1:**
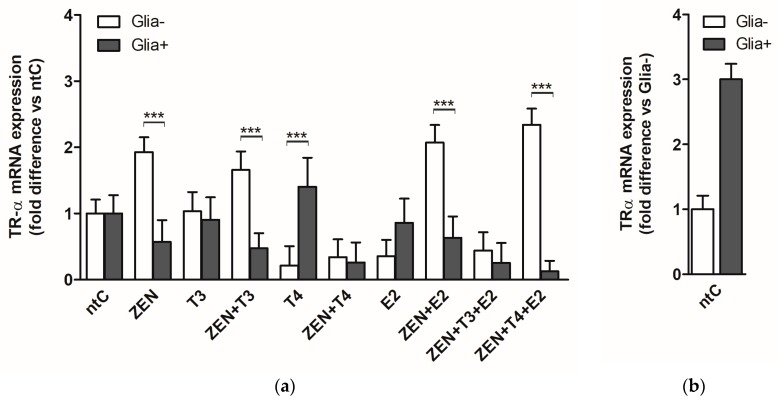
TRα mRNA expression in cerebellar granule cells in the absence, Glia−, or presence of glial cells, Glia+; treated with zearalenone (ZEN), and/or triiodo-thyronine (T3), thyroxine (T4) and 17β-estradiol (E2) for 6 h. (**a**) Relative expression level of the TRα gene was analyzed by qRT-PCR and normalized to the average of the control gene β-actin. Shown P-values were calculated as follows: Glia+ compared to Glia− (***) *p* < 0.001 in each treatment group. (**b**) Relative expression of TRα mRNA in non-treated controls normalized to β-actin (*p*-value not shown). The data shown here are the mean ± standard deviation (SD) of at least three independent experiments (*n* = 6 per treatment).

We first examined the transcription rates of the TRα gene for which we quantified the TRα mRNA accumulation following 6 h treatment with the different hormones (T3, T4, E2) and ZEN alone or in combination with these hormones (Figure 1).

In glia reduced granule cultures (Glia−), ZEN increased the mRNA levels of TRα compared to non-treated control (ntC), *p* < 0.001. No changes in the TRα expression were observed applying T3 hormone, whereas T4 and E2 led to a significant drop in TRα expression compared to ntC (*p* < 0.01). T3 in combination with ZEN increased TRα mRNA levels comparatively to those induced by ZEN alone and increased significantly compared to T3 administered alone. TRα mRNA expression levels in case of T4 + ZEN were similar to the decreased levels (5.6 fold drop compared to ntC) elicited by T4 alone and compared to ZEN. ZEN + E2 induced high expression levels even compared with that of ZEN and as such, significantly higher compared to reduced expression levels observed with E2 hormone alone. Concurrent administration of ZEN, T3 and E2 led to decreased mRNA levels, similar to the low levels observed in the case of E2 treatment, whereas ZEN + T4 + E2 combination resulted in high TRα expression levels comparable to those observed in the case of ZEN + E2 combination, thus significantly higher compared to T4 and E2 induced effects (*p* < 0.001).

In the presence of glial cells (Glia+ cultures), ZEN-induced TRα mRNA levels did not alter significantly from those detected in the ntC. Similarly, no changes were observed in the case of T3 and E2 treatments; however, a significant increase was detected in the case of T4 treatment compared to ZEN (*p* < 0.01). ZEN in the presence of T3, T4, and E2 had a negative effect on TRα expression. The lowest mRNA levels were observed in the case of ZEN + T3 + E2 and ZEN + T4 + E2 combinations. 

Considering the two primary culture systems, TRα mRNA levels altered significantly in Glia− cultures compared to Glia+, with a significant increase in Glia− following ZEN treatment (*p* < 0.001). High expression of TRα mRNA was observed in the case of ZEN used in combination with T3, E2, and T4 + E2 in Glia−. A reverse expression pattern was observed in the case of T4 treatment resulting in elevated mRNA levels in the Glia+ compared to the low levels of mRNA detected in the Glia− (*p* < 0.001). 

#### 2.1.2. TRα Protein Levels

**Figure 2:**
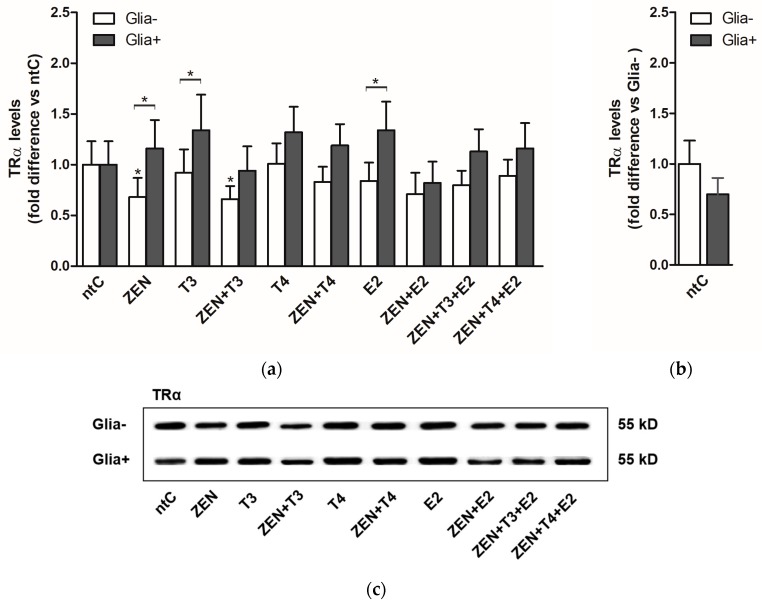
TRα protein expression levels in cerebellar granule cells in the absence, Glia−, or presence of glial cells, Glia+; treated with zearalenone (ZEN), and/or triiodo-thyronine (T3), thyroxine (T4) and 17β-estradiol (E2) for 18 h, examined by Western blotting; (**a**) Shown P-values were calculated: compared to ntC (above and next to the bars) and Glia+ compared to Glia− (*) *p* < 0.05 (above braces) in each treatment group; (**b**) Expression of TRα protein in non-treated controls normalized to Glia− (*p*-value not shown). All data represents the mean ± SD of at least three independent experiments (*n* = 6 per treatment); (**c**) Representative Western blot images.

In Glia− neuronal cultures, the protein levels did not reflect the transcriptional activity detected on mRNA levels. As opposed to high transcriptional rates, a significant decrease in protein levels was observed following ZEN and ZEN + T3 treatments (Figure 2). Compared to ntC, no changes were observed in the Glia+, regardless of the applied treatment. However, significant changes were detected between the two culture systems (Glia− and Glia+) in the case of ZEN, T3, and E2 treatments. 

### 2.2. Expression of Thyroid Hormone Receptor Beta (TRβ)

#### 2.2.1. TRβ mRNA Expression

**Figure 3:**
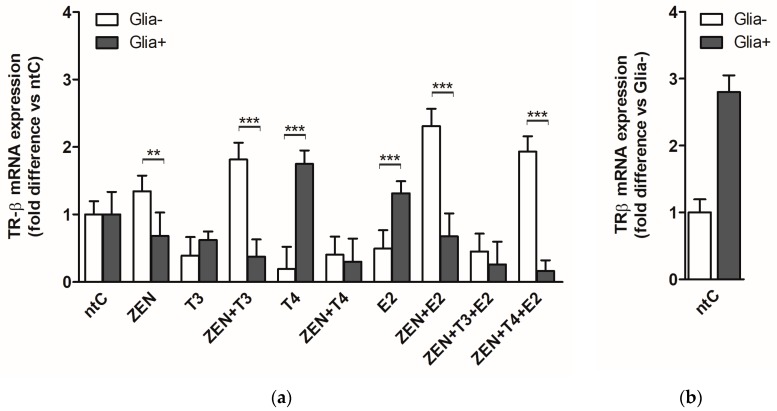
TRβ mRNA expression in cerebellar granule cells in the absence, Glia−, or presence of glial cells, Glia+; treated with zearalenone (ZEN), and/or triiodo-thyronine (T3), thyroxine (T4) and 17β-estradiol (E2) for 6 h. (**a**) Relative expression level of the TRβ gene was analyzed by qRT-PCR and normalized to the average of the control gene β-actin. Shown *p*-values were calculated: Glia+ compared to Glia− (**) *p* < 0.01, (***) *p* < 0.001 in each treatment group; (**b**) Relative expression of TRβ mRNA in non-treated controls normalized to β-actin (*p*-value not shown). The data shown here are the mean ± SD of at least three independent experiments (*n* = 6 per treatment).

In Glia−, ZEN at 0.1 nM concentration did not alter the TRβ mRNA expression. T3 and T4 exerted an inhibitory effect on TRβ expression reducing the mRNA levels (*p* < 0.01). Similar effect was observed after E2 treatment. ZEN in combination with T3 increased the TRβ expression levels compared to ZEN. Similarly, ZEN applied with E2 increased the mRNA levels (*p* < 0.01), while T4 + ZEN did not enhance mRNA levels compared to the low levels induced by T4 alone. Concurrent treatment with ZEN, T3, and E2 had a decreasing effect on TRβ expression resulting in low levels of mRNA as observed in the case of T3 and E2 treatments. Combination of ZEN, T4 and E2 resulted in high TRα mRNA levels comparable to those observed after ZEN + E2 combined treatment and opposite to the low levels induced by T4 and E2 (Figure 3).

In Glia+, ZEN had no effect on TRβ expression similarly to T3 and E2 treatment, whereas T4 induced a three-fold increase in TRβ expression. ZEN in combination with T3 did not change significantly the expression observed in the case of T3. Treatment with ZEN + T4 inhibited the expression of TRβ causing a 5.6-fold decrease compared to T4. Zen + E2 combination also decreased the mRNA expression levels compared to E2 (*p* < 0.05). ZEN + T3 + E2 induced combined effects were similar to those observed with T3; however, compared to E2 a significant decrease of expression could be observed (*p* < 0.01). In a similar fashion, low levels of mRNA expression were observed with ZEN + T4 + E2 combination compared to ZEN and T4.

With regard to the glia effects, significant differences in expression levels were observed between Glia+ and Glia− after ZEN, ZEN + T3, ZEN + E2, and ZEN + T4 + E2 treatments, with increased mRNA levels in Glia− compared to the low levels detected in Glia+. A reverse expression pattern was observed after T4 treatment with increased mRNA levels in the Glia+ group and low levels in Glia− (*p* < 0.001).

#### 2.2.2. TRβ Protein Levels

**Figure 4:**
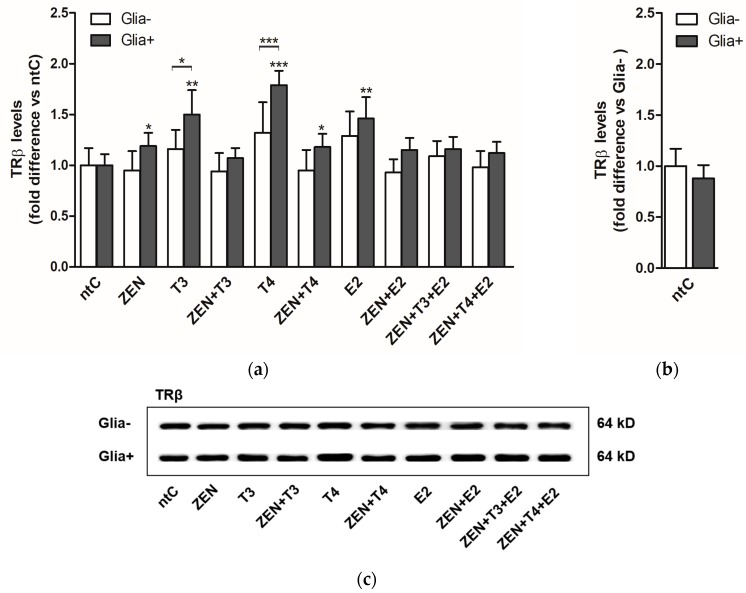
TRβ protein expression levels in cerebellar granule cells in the absence, Glia−, or presence of glial cells, Glia+; treated with zearalenone (ZEN), and/or triiodo-thyronine (T3), thyroxine (T4) and 17β-estradiol (E2) for 18 h, examined by Western blotting. (**a**) Shown P-values were calculated: compared to ntC (above and next to the bars) and Glia+ compared to Glia− (*) *p* < 0.05, (**) *p* < 0.01, (***) *p* < 0.001 (above braces) in each treatment group; (**b**) Expression of TRβ protein in non-treated controls normalized to Glia− (*p*-value not shown). All data represents the mean ± SD of at least three independent experiments (*n* = 6 per treatment); (**c**) Representative Western blot images.

ZEN treatment affected the translation of TRβ, resulting in increased protein levels (*p* < 0.05) in the presence of glial cells (Figure 4). Treatments with T3 and T4 alone also induced translation with significantly high protein levels (*p* < 0.01 and *p* < 0.001, respectively) compared to ntC in the same experimental group. In Glia−, no significant changes were observed in protein levels regardless of the applied treatment. 

## 3. Discussion

Numerous in vitro and in vivo studies demonstrated that ZEN can competitively bind to estrogen receptor and as such, act as an endocrine disruptor leading to a variety of physiological consequences [39,40]. According to a European Union assessment published in 2004, 32% of the examined crop samples were contaminated with ZEN. This result signifies the importance of ZEN effects both in domestic animals and humans. In a brief overview of the proven and/or potential biological effects of ZEN the first observation that should be alarming is that besides certain interspecies differences, ZEN effects are reported from the same organs of various species, including humans as well. Although ZEN, as an estrogenic compound is likely to alter estrogen’s effects throughout the organism, ZEN seems to affect some organs more than others in more than one species. These are the immune system, the liver, kidney, and the reproductive system in both sexes. It is well known that ZEN decreases the number of blood cells in rats, pigs, and humans [41] and also decreases the number of platelets [42], may cause apoptosis and cell necrosis in blood cell generating organs [43]. ZEN also plays a role in the development of myelophibrosis and osteoporosis [44]. Since Zen can bind to ERs, and ERs are expressed in T- and B-lymphocytes, monocytes, and macrophages [45,46,47], these cells are all likely targets of ZEN. Thus, it is not surprising that chronic treatment of ovariectomized rats with ZEN resulted in atrophy of the thymus and a decrease of B-cells in the spleen [48]. Similar destructing effects were reported from mouse experiments, where ZEN decreased the number of lymphocytes, B- and T-cells, and also decreased the level of IgG and IgM [49]. Consonantly, ZEN decreased white blood cell numbers in chicken [50,51], cattle [52,53] and humans [54,55]. Altogether, these data indicate the ubiquitous immune suppressive effects of ZEN.

It has been reported that, after ingestion of contaminated feed, ZEN was detectable in the liver of the examined species, such as fish [56], turkeys [57], rabbits [58], rats, and pigs [42,59]. Because of the cytotoxic effects of ZEN in the liver [60], ingestion of this endocrine disruptor (ED) should be considered as a threat to hepatic functions in humans as well. Although little or no report can be found from human studies, ZEN alters the level and activity of some liver enzymes (such as aspartate aminotransferase (AST), alanine aminotransferase (ALT), gamma-glutamyl transferase (GGT), and lactate dehydrogenase (LDH)) in the examined species, including rabbits [58], rats [59,61], and pigs [44]. In addition to the adverse effects of ZEN on liver functions, the metabolization of ZEN continues in the kidneys where it exerts further destructive effects. Indeed, Zen was shown to cause kidney malfunctions in more species [62,63]. Altogether, ZEN effects observed in the liver and kidney suggest that these effects are ubiquitous among species and therefore may decrease the detoxicating capacity of the human organism as well.

ZEN, as most of the EDs, effects the reproductive system as well. These ED effects are broadly documented among the affected organ systems, and in this respect not only females but males are targets of ZEN (and other EDs) as well [61,64,65]. ZEN can penetrate through the blood brain barrier (like other EDs [66]), and modify hypothalamic and pituitary functions in all examined species. It is well known that ZEN and other EDs may exert adverse effects on central, as well as peripheral organs of the reproductive system. Although the human exposure can be high in certain areas, the role of ZEN in human fertility disruption and related diseases are not thoroughly investigated, yet recent evidence suggests a high impact of different xenoestrogens during critical periods of embryonic development on gene expression and unexpected effects on metabolism [67]. It has been also reported that ZEN and its metabolites promote the development of different hormone-dependent tumors in humans [8]. Additionally, ZEN and its analogues might have potential applications as drugs in estrogen replacement therapies [68]. Different cellular mechanisms have been evaluated to elucidate the endocrine and neurotoxic effects of ZEN on brain function [14]; however, no data are available regarding ZEN’s modulatory effect on thyroid receptor (TRs) expression in the cerebellum. Several studies reported that different chemicals can bind to TRs and may evoke complex TH signaling events [69,70], therefore, we focused our research on ZEN-induced TRα and TRβ mRNA and protein expression profiles in primary cerebellar granule cells. Since THs and steroid hormones are highly involved in cerebellar development and plasticity, their modulatory effects on ZEN-induced expression was also assessed by applying physiological concentrations of T3 and T4 hormones, as well as E2 in the presence of ZEN. Our group previously reported the regulatory role of glia on TR and estrogen receptor (ER) activity [23], thus standardized culture conditions were applied in this study in order to obtain comparable results in the glia effect on ZEN induced TRs expression. In the applied experimental setup, the granule cells were well dispersed without aggregates or cell clumps. Thus, ZEN and hormonal treatments were more uniform within the culture, and glia–neuron contacts were balanced. 

A direct and receptor subtype selective effect of ZEN on TRα receptor expression was confirmed in the primary cerebellar granule neurons in the present study. In our experimental setups, the applied culture conditions had a high impact on TRα transcription and translation profiles following ZEN or hormonal treatment. The increased TRα mRNA expression was measured in the relatively clear neuronal cultures (Glia−) indicating a selective and direct binding potential of ZEN to TRα binding sites. Moreover, ZEN-induced mRNA expression was abolished in the presence of glial cells (Glia+) suggesting a modulatory effect of glia on ZEN-induced transcription. Glia receptivity and sensitivity to THs were previously demonstrated in several studies indicating a direct effect of THs on astrocyte and microglia development, maturation, and differentiation [30]. The absence of TRα and TRβ transcriptional response to ZEN in the presence of glial cells can be attributed to a direct interaction between ZEN and glia, most probably by an active uptake and clearance mechanisms exercised by glia [71]. Since the two neuronal culture systems, Glia+ and Glia−, clearly differ in the number and development of glial cells, the observed modulatory effects exhibited by glia can be accounted for by differences in the neuronal cell culture conditions. Hence, no increase in the transcription of TRα was observed following ZEN or T3 and E2 treatments despite of the high (three-fold) basal expression of TRα in the non-treated Glia+ controls, compared to the Glia− control group (Figure 1a,b). Furthermore, the presence of glial cells did not impact TRα and TRβ mRNA levels in response to T3 and E2 hormones as observed in the absence of glia; however, glial cells did have a positive effect on expression of both thyroid receptors following T4 treatment. Intriguingly, the presence of glia appeared to have an inhibitory effect on TRα and TRβ transcription after co-administration of ZEN with T3, T4, and/or E2 significantly decreasing the basal transcription. It should be noted at this point that these neuronal cultures are sustained in a serum and steroid free environment and as such, it might induce certain intracellular adaptations under the applied experimental conditions, resulting in higher transcriptional activities of the ligand-bound receptors. Moreover, dependence of glial cells on THs for their normal function might be increased in the applied experimental system, causing a quick clearing of the available hormones and of ZEN and inhibiting their binding propensities to the nuclear receptor binding site. Interestingly, in the absence of glial cells, the effects of ZEN on TRs transcription were also highly potentiated by the presence of THs or estrogen, induced either by a competition in the co-activator complexes formation or/and to thyroid hormone response elements or by a direct enhancing effect when both ligands are present involving different non-genomic signaling pathways [72] via transmembrane integrins [73] or receptor tyrosine kinases [74]. This cumulative effect on TR expression elicited by ZEN co-administered with T3 and E2 was abolished and inhibited in the presence of glial cells. No apparent disturbances in cell viabilities were observed after ZEN treatment, however, glial cells present in the granule neuronal cultures might represent a selective glia population more susceptible to ZEN and to ZEN-induced clearance and metabolic inhibition [75,76,77].

On a translational level, the presence of glia did not result in significantly higher protein levels with respect to TRα. Moreover, in the absence of glia the protein expression was significantly decreased in ZEN and ZEN + T3 treatment groups, in contrast to the increased mRNA expression levels induced by ZEN and hormones observed in the same experimental group. It is well-known that protein expression is determined by many factors not only at mRNA level, and many times the protein levels correlate poorly with the mRNA levels [78,79]. In line with the aforementioned statement, on a protein level, the changes in the receptor expression profiles varied less compared to those observed at the mRNA level where the applied hormones and ZEN had a higher impact on expression profile of TRα, which can indicate the impact of growth conditions on protein concentrations in steady-state cell neuronal populations. 

It has been shown that TRα is the most abundant thyroid receptor in the developing brain and the two receptors are highly heterogeneous in their expression throughout the different brain regions. These changes might be also reflected in their responsiveness in our experimental system since TRβ expression was not influenced by ZEN at mRNA level opposite to TRα expression. Interestingly, the lack of TRβ mRNA expression was contravened at protein level since a small but significant increase in TRβ protein expression was induced by ZEN, suggesting the increased expression and functionality of TRβ in mature cerebellar neurons [80]. This was also reflected by the significant increase of TRβ protein levels induced by T3, T4, and E2. It should be noted that the increased protein levels caused by ZEN and hormones were only observed in the presence of glia, proving their modulatory effect, and in part, explaining the TRβ expression in response to T4; however, a direct modulation of TRs by T4 was also proposed by other studies [81]. Both TRα and TRβ receptors have been identified in different types of astroglia and microglia [33,82] and their differential expression might also contribute to the increased protein expression observed in Glia+ cultures.

Glial cells induced receptor expression modulation as clearly observed in the case of T4-induced bidirectional transcriptional rates between the Glia+ and Glia− cell cultures for both TRα and TRβ receptors. T4-induced high levels of mRNA in Glia+ culture conditions may imply type-2 deiodinase activity of glial cells, converting T4 to T3 and mediated in a paracrine way; whereas, in Glia− cultures the activity of T4 was completely diminished, marking a silencing action on TRs expression, probably due to the absence of glial cells and thus lacking the conversion of inactive T4. A non-genomic silencing effect can be also postulated in the absence of glia and sole T4 treatment [83]. The evidence for a paracrine interaction between astrocytes and neurons was also demonstrated by other in vitro studies where T3 produced by D2 activity in glial cells promoted gene expression in neurons [84]. 

A large body of evidence demonstrates the importance of membrane transporters in the active transport and uptake of thyroid hormones through the cell membranes [85,86]. The most specific transporters for THs are organic anion-transporting polypeptides (OATPs), L-type amino acid transporters (LAT1 and LAT2), and monocarboxylate transporters 8 (MCT8) [87,88,89] facilitating the uptake of T4 and T3 into neurons and glia in a hormone-specific fashion. These transporters play important roles in the neuron–glia interactions mostly proved in neuron-glia co-cultures [90] and they are potential targets for different endocrine disruptors [91]. Modulation of TRs expression by glial cells might be facilitated by a direct neuron–glia metabolic coupling or by direct uptake of ZEN into the glial cells and thus buffering and reducing the applied ZEN concentration. Moreover, ZEN along with other mycotoxins were proved to directly interact with organic anion transporters (OATPs) and organic cation transporters (OCTs) as potential transport substrates for these transporters and thus modulating the uptake of other substances [92]. 

We also observed that the active form of thyroid hormone, T3, applied alone did not alter TRα mRNA levels and had a negative effect on TRβ transcription levels; however, the presence of glia cells induced high TRβ protein levels. These findings also underline the importance of glial cells in the modulation of hormone responses in neuronal cell cultures and CNS. 

Our findings suggest that ZEN, aside from possessing estrogen agonist properties [93], can induce TRs expression that is further affected and modulated by various factors such as the presence of glia cells, thyroid, and steroid hormones. Additionally, the observed changes in transcription and translation rates in response to ZEN treatment might be induced by certain cellular adaptations to the applied experimental conditions and by the loss of tissue integrity, such as the absence of extracellular matrix proteins, integrins, and certain glycoproteins that play role in neuron–glia communication [94,95]. 

## 4. Materials and Methods 

### 4.1. Antibodies, Reagents, and Materials

TRα and TRβ primary antibodies were purchased from Abnova (Budapest, Hungary). Secondary antibodies were from Vector Laboratories. Protease inhibitor cocktail, β-d-arabinofuranoside, 17β-estradiol, 3,3′,5-triiodo-l-thyronine, l-thyroxine, and zearalenone were purchased from Sigma-Aldrich (Budapest, Hungary). Culture media and TRI reagent were from Invitrogen (Carlsbad, CA, USA). Penicillin/streptomycin and heat-inactivated fetal bovine serum were purchased from GIBCO (Budapest, Hungary). Bicinchoninic acid (BCA) kit from Pierce (Rockford, IL, USA). Immobilon Western chemiluminescent HRP substrate was from Merck Millipore (Darmstadt, Germany). 

### 4.2. Animals

Sprague Dawley rats were purchased from TOXI-COOP Zrt. Budapest, Hungary and maintained in the animal facility of the University of Veterinary Medicine, Budapest. Animals were kept under standard laboratory conditions, allowed free access to food and water, and maintained on a 12/12-h light/dark cycle until the time of sacrifice. The animals were treated according to the EC Council Directive of 24 November 1986 (86/89/EEC) and all procedures were reviewed and approved by the local ethical committee (Animal Welfare Board at University of Veterinary Medicine and regional animal welfare authority, ID: PE/EA/1252-6/2016, date: 05/2016, Pest Megyei Kormányhivatal).

### 4.3. Preparation and Culture of Cerebellar Granule Cells 

Dissociated cerebellar cells were prepared from seven-to-nine day-old rats from both sexes—since no gender differences were observed in the present and in previous studies—and were obtained by the method previously described with small modifications [96]. The removed cerebella were dissociated without enzymatic treatment by repeated trituration. Triturated cells were filtered through a nylon cell strainer by gravity (pore size 40 µm) to remove large, non-dissociated cell clumps and non-neuronal cells. Cells were seeded in poly-L-lysine pre-coated petri dishes at densities of 2300–2700 cells/mm^2^ and maintained in culture for seven days (37 °C, 5% CO_2_) in serum and steroid free Dulbecco’s modified Eagle medium supplemented with 5 µg/mL insulin, 5 µg/mL transferrin, 5 µg/mL selenium, and 20 mM KCl for mild depolarization and survival of the cells [22,97,98]. The resulting cultures consisted of non-clustered, granule cell type neurons (~95%).

### 4.4. Treatments

**Figure 5:**
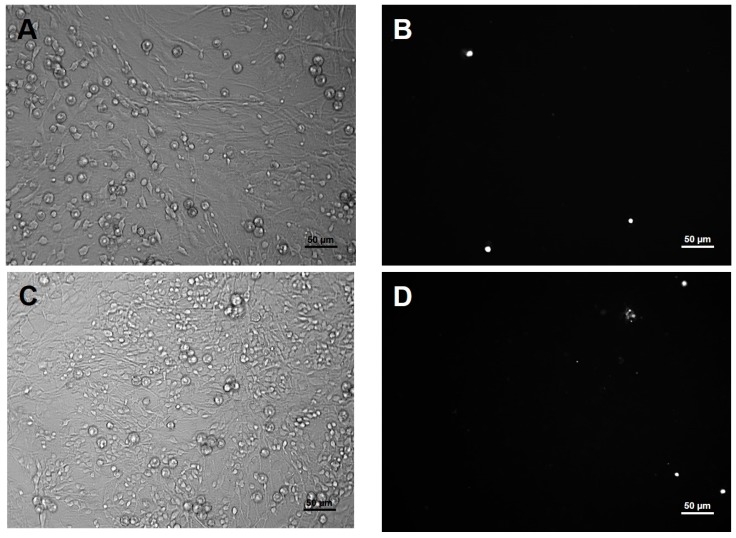
PI staining was used to evaluate cell viability following ZEN treatment. (**A**,**B**) Non-treated, Glia+ control group, (**C**,**D**) ZEN treated Glia+ group. PI positive cells were detected by fluorescence microscopy using a Texas red filter set.

For analysis of mature primary cerebellar cells a final concentration of 10 μM cytosine β-d-arabinofuranoside (Ara-C; Sigma-Aldrich Ltd., Budapest, Hungary) was added 24 h after seeding to inhibit the proliferation of non-neuronal cells (Glia− experimental groups). Ara-C non-treated experimental groups of neuronal cultures were also included in the study (Glia+ experimental groups). The presence of glial cells in the glia positive cultures (Glia+) was evaluated as described earlier [23]. Cell viability was measured using propidium iodide (PI) staining (Figure 5).

Cells were treated after seven days of growth in culture with the following hormones at physiologically relevant concentrations: 116 nM 17β-estradiol (E2), 0.92 nM 3,3′,5-triiodo-l-thyronine (T3), 65 nM l-thyroxine (T4) and/or the endocrine disruptor zearalenone (ZEN). ZEN concentration was set to 0.1 nM according to literature [2,14] as the expected brain concentration after chronic exposure to this mycotoxin. Treatments were applied for 6 h for qRT-PCR and 18 h for Western blot studies before cell harvesting. The 6 h incubation time for mRNA expression was chosen based on our previous experimental readouts and the sensitivity of the applied neuronal cell culture system. For the detection of changes in protein translation a longer incubation period was chosen considering that shorter incubation periods gave no consistent responses. The 6 and 18 h of treatment times was also based on the fact that translation is downstream to transcription and requires a longer time to manifest in realistic amounts. Non-treated controls were included in both Glia+ and Glia− experimental groups. In case of dimethyl sulfoxide (DMSO) vehicle, it was used at 0.1% concentration and had no effect on cultured cells. Experiments were repeated at least five times with six parallel dishes per treatment. 

### 4.5. Revers Transcription- and Quantitative-RT-PCR

Total RNAs were extracted using TRI reagent following the manufacturer’s protocol (Invitrogen, Carlsbad, CA, USA). 3 μg of total RNA was reverse transcribed by RT-PCR in a final volume of 30 μL using Red Taq Polymerase (Sigma, Budapest, Hungary) and oligo(dt) primers. cDNA was stored at −80 °C and 2 μL was analyzed in triplicate by qRT-PCR (Master SYBRGreen, Hoffmann-La Roche, Basel, Switzerland) in a LightCycler 2.0, F. device (Hoffmann-La Roche, Basel, Switzerland) using β-actin gene as endogenous control for normalization of the data [99]. The primer sequences used for amplification of TRα and TRβ were as previously published by Billon [100] and Kariv [101], respectively. qRT-PCR cycles and controls were planned according to the manufacturer’s instructions and were optimized for the primer pair. Cycling parameters were as follows: one cycle at 95 °C for 30 s for enzyme activation, 45 cycles of initial denaturation at 95 °C, annealing (primer dependent) and extension cycles at 72 °C, one cycle at 65 °C for 15 s and 95 °C for 1 min. Amplified products were identified by agarose gel electrophoresis, melting point, and sequence analysis (Applied Biosystems ABI 3100 Genetic Analyzer, Agricultural Biotechnology Center, Gödöllő, Hungary). Real-time fluorescent measurements were taken at every cycle and change in threshold cycle (Δ*C*_t_) was calculated. Real-time PCR threshold cycle (*C*_t_) data were analyzed using the REST-XL software version 2.0 (GenEx—BioMcc, TUM, München, Germany) [102]. Cycle threshold values were normalized to those of β-actin. The relative expression ratios of mRNA (fold changes) were calculated using the 2^−ΔΔ*C*t^ method.

### 4.6. Western Blot Analysis

Cerebellar cells grown on petri dishes were harvested by washing with ice-cold PBS and collected by centrifugation as previously described [96]. Total cell lysates were prepared by homogenization and sonication for 10 s a total of three times in lysis buffer (20 mM Tris-HCl pH 7.5, 150 mM NaCl, 1 mM PMSF, 1 mM EGTA, 1 mM EDTA, 2.5 mM sodium pyrophosphate, 1 mM β-glycerol phosphate; and 1 mM Na_3_VO_4_ plus 1 mg/mL Pefabloc, 10 μg/mL leupeptin, 10 μg/mL pepstatin, 1 μg/mL aprotinin; and 1% Triton X-100, 0.05% sodium deoxycholate). Protein concentrations were determined with a BCA protein assay kit (Pierce, Rockford, IL, USA). Equal amounts (10 μg/lane) of protein were loaded onto 8–12.5% (*w*/*v*) sodium dodecyl sulfate-polyacrylamide gels. Separated proteins were blotted onto polyvinylidene difluoride (PVDF) membranes (Merck Millipore, Budapest, Hungary). Membranes were blocked in 5% (*w*/*v*) nonfat milk for 1 h diluted in Tris-buffered saline supplemented with 0.1% (*v*/*v*) Tween-20 (TBST) followed by an incubation with primary antibodies anti-thyroid hormone receptor α1 (c-erbA-1, PAB11276), dilution 1:1000 and anti- thyroid hormone receptor β1 (c-erbA-2, PAB11277) dilution 1:500 at 4 °C overnight, and then with secondary peroxidase coupled anti-rabbit or anti-mouse antibodies (1:2000, Vector laboratories, Hungary) at room temperature for 1 h. After exposure to Amersham hyperfilm MP (GE Healthcare, Budapest, Hungary) intensities were analyzed using Image J software (NIH—open source). All data that have been presented are representative of at least three independent experiments (*n* = 6 per treatment).

### 4.7. Data Analysis

All data that have been presented are representative of at least three independent measurements. Statistical analyses were conducted using Excel (Microsoft, Microsoft Co., Redmond, WA, USA) and GraphPad Prism version 4 (GraphPad Software, San Diego, CA, USA), by means of two-way ANOVA with Bonferroni post-tests and/or unpaired *t*-tests. Statistical analyses were carried out by the Department of Biomathematics, University of Veterinary Sciences, Budapest, Hungary.

## 5. Conclusions

In conclusion, here we show that ZEN directly applied to primary cultures of cerebellar granule neurons displays receptor subtype selective effect on transcription of TRα and TRβ. The ZEN induced transcriptional profiles of TRs is highly influenced by the applied experimental conditions (presence or absence of glia cells) and the hormonal profile. Glial cells exhibit a direct modulatory effect on ZEN induced TRα and TRβ transcription, displaying mostly a silencing effect on basal mRNA transcription, which translated to in vivo might anticipate the potential regulatory effect of ZEN on early postnatal differentiation and development of cerebellum with potential pathological relevance, since TRα and TRβ are closely related to cellular differentiation (neuron and glia), maturation, and brain development. The presence of physiological amounts of THs or E2 also modulates ZEN induced transcriptional rates assuming combined inhibitory and/or potentiating effects of ZEN on receptor transcription in accordance with the experimental conditions. Thus, the regulatory role of glial cells and of endogenous hormones agrees with earlier observations suggesting that neuron-glia communication and hormonal profile impact the effects induced by different endocrine disruptors at molecular levels [23,37]. These findings demonstrate the importance of experimental conditions applied in the study of endocrine disruptors mediated molecular effects on nuclear receptor transcription and translation and as such, might be taken into consideration for further studies with other endocrine disruptors or members of the mycotoxin family.

## Abbreviations

ZEN	zearalenone
TRα, TRβ	thyroid receptor alpha, thyroid receptor beta
T4	l-thyroxine
T3	3,3′,5-triiodo-l-thyronine
E2	17β-estradiol
